# Investigating the effect of type of fish and different cooking methods on the residual amount of polycyclic aromatic hydrocarbons (PAHs) in some Iranian fish: A health risk assessment

**DOI:** 10.1016/j.fochx.2023.100789

**Published:** 2023-07-08

**Authors:** Gholamali Sharifiarab, Mohammad Ahmadi, Nabi Shariatifar, Peiman Ariaii

**Affiliations:** a Department of Food Hygiene, Ayatollah Amoli Branch, Islamic Azad Uneversity, Amol, Iran; b Department of Environmental Health Engineering, School of Public Health, Tehran University of Medical Sciences, Tehran, Iran; c Department of Food Science and Technology, Ayatollah Amoli Branch, Islamic Azad Uneversity, Amol, Iran

**Keywords:** Cooking methods, BaP (Benzo[*a*]pyrene), Gas chromatography-mass spectrometry (GC–MS), Fish, Magnetic solid phase extraction (MSPE)

## Abstract

The aim of this study was to assess the level of PAHs and associated health risks in different types of fish cooked with different methods, using the MSPE-GC/MS technique (magnetic solid phase extraction with gas chromatography/mass spectrometry). The limits of detection (LODs), limits of quantification (LOQs) and recovery percentages ranged from 0.1 to 0.63 μg/kg, 0.3–1.89 μg/kg, and 93.7 to 102.6%, respectively. The results showed that the mean of ƩPAHs in all samples was 20.31 ± 6.60 µg/kg. Additionally, PAH4 and BaP levels in all samples were 4.58 ± 1.40 and 1.08 ± 0.36 µg/kg, respectively, which were below the European Union (EU) standard level (12 and 2 µg/kg, respectively). The results showed that among 5 types of fish, starry sturgeon had highest average total PAHs (13.24 ± 1.84 µg/kg), while Caspian Sea sprat had the lowest average total PAHs (1.24 ± 0.8 µg/kg). In terms of cooking methods (charcoal-grilled fish, fried fish and oven-grilled fish), charcoal-grilled fish had the highest average total PAH level at 25.41 ± 7.31 µg/kg, while the lowest average total PAH was found in the raw fish sample at 16.44 ± 4.63 µg/kg. The Monte Carlo Simulation was used to determine the 95% ILCRs (Incremental Lifetime Cancer Risk) due to ingestion of fish. The results showed that the ILCR for adults was 2.85E-9, while for children it was 1.32E-8. Therefore, based on these findings, it can be concluded that the consumption of fish cooked with different methods does not pose a risk to human health in terms of the amount of PAHs (ILCR < 1 × 10^−4^).

## Highlights

• In all samples, PAH4 and BaP were less than the EU (European Union) standard level.
• Starry sturgeon fish had highest and Caspian Sea sprat had lowest mean total PAHs.
• Charcoal grilled fish had the highest and raw fish had the lowest mean total PAHs.
• The consumption of fish does not pose a risk to human health.

## 1 Introduction

In recent years, an increase in persistent organic pollutants (POP) has been observed in both terrestrial and aquatic environments. These pollutants, such as Polycyclic aromatic hydrocarbons (PAHs), polychlorinated biphenyls (PCBs) and other compounds are among the POP, can be found in exist in water, soil and air and can enter the food chain directly or indirectly (Moazzen et al., 2022, Shariatifar et al., 2021, Shariatifar et al., 2022, Sharifiarab et al., 2022).

A healthy diet is crucial for maintaining good health, and it should contain adequate amounts of nutrients while limiting chemical pollutants and preventing exposure to pathogenic microorganisms. Seafood, particularly fish, is an essential source of polyunsaturated fatty acids (PUFAs), including omega-3, vitamins, and proteins. However, the potential risks associated with frequent consumption of fish and other seafood due to exposure to chemical pollutants cannot be ignored. Despite this concern, the consumption of these foods is highly recommended to prevent various diseases, including cardiovascular diseases (Magalhães et al., 2022, Shariatifar et al., 2022, Udofia et al., 2021).

PAHs are a large class of organic compounds containing two or more fused aromatic rings. These compounds ubiquitous environmental pollutants generated primarily during the incomplete combustion of organic materials (gasoline, charcoal/wood, coal, benzene, oil, etc.). The foremost sources of PAHs (anthropogenic) include associated activities in exhaust of a motor vehicle, residential heating, coke and aluminum production, catalytic cracking towers and petroleum refineries, coal-tar pitch and production of asphalt and also liquefying plants and gasification of coal (Naghashan et al., 2022, Shariatifar et al., 2022). Most PAHs are anthropogenic and released into the environment, but sometimes they are also released by natural disasters, volcanic activity, forest fires, and oil or coal explosions. Because of this, PAHs can spread through soil, water, air, and food (Kiani et al., 2019, Moazzen et al., 2022, Sharifiarab et al., 2022).

According to the statement of WHO (World Health Organization), they are absorbed through breathing, digestive system (gastrointestinal) and skin. Based on the type of diet in different regions of the world, people receive PAH analytes in different amounts. According to the researcher findings, in animals, thirty to fifty percent of PAH analytes are absorbed via the GI tract and finally, they are processed in the human liver. It is a major concern for health of human when dioepoxide metabolism is activated, since it leads to mutation and replication of DNA (Dutta et al., 2022, Ferrante et al., 2018, Khalili et al., 2023).

The USEPA (United State Environmental Protection Agency) introduced 16 PAH analytes including (anthracene (An), dibenzo[*a*,*h*]anthracene (DahA), benzo(a)anthracene (BaA), benzo[*g*,*h*,*i*]perylene (BgP), fluoranthene (Fla), chrysene (Chr), indeno[1,2,3-*cd*]pyrene (IcdP), benzo(k)fluoranthene (BkF), acenaphthylene (Acy), benzo(a)pyrene (BaP), fluorene (Fle), acenaphthene (Ace), naphthalene Napphenanthrene (Ph), benzo(b)fluoranthene (BbF), and pyrene (Py)). Considering food safety, BaP analyte can be used to identify PAH analytes that contribute to food cancer. Many studies were shown, PAH exposure is positively associated to numerous illnesses such as cancer of stomach, biochemical and cytogenetic alteration, and alteration in the lungs including DNA damage. According to the EU (European Union) standard levels in fish samples, BaP and PAH4 should not exceed 2 and 12 μg / kg, respectively (Jafarabadi et al., 2020, Qian et al., 2022).

According to many studies, a large number of foods are contaminated with PAH compounds such as beverages (such as water, coffee and tea), vegetables/fruits, smoked foods, meat and related products, grains, fats and oils, fish and seafood, as well as milk and related products. PAH analytes can enter raw foods and cooked foods in various ways during cooking with incomplete combustion of wood, gas, etc. In addition, the accumulation of PAH analytes in fish and seafood can be affected by many reasons, including the physiological characteristics of the fish (such as weight, age, size, etc.), the water of the fish breeding area and the food that the fish consumes, whether farmed or wild, etc. (Gorji et al., 2016, Shariatifar et al., 2021, Shariatifar et al., 2022).

Today, different methods are used to extract and analyze different pollutants in food and non-food matrices (Awual, 2015; M. M. Hasan et al., 2021, Kubra et al., 2021, Kubra et al., 2021, Salman et al., 2021). In the current study, the MSPE/GC–MS method, which is a simpler and less expensive method, was used.

Since comprehensive and complete research on the amount of PAH analytes in Iranian fish with different cooking methods has not been done and considering the high consumption of fish and seafood in Iran, it seems necessary to conduct this study. Therefore, the aim of the present study was to investigate the amount of 16 PAH compounds (using MSPE-GC/MS technique) and their human health risk (by Monte Carlo Simulation) in 5 types of fish consumed by Iranians ((trout (*Oncorhynchus mykiss*), Caspian kutum (*Rutilus kutum*), carp (*Cyprinus carpio*), starry sturgeon (*Acipenser stellatus*) and Caspian Sea sprat (*Clupeonella Cultriventris*)), as raw form and cooked forms (fried fish, grilled fish on the charcoal and grilled fish on the gas oven).

## 2 Materials and methods

### 2.1 Fish sampling

Fish samples for determining the amount of PAH compounds included 5 commonly used types (trout (*Oncorhynchus mykiss*), Caspian kutum (*Rutilus kutum*), carp (*Cyprinus carpio*), starry sturgeon (*Acipenser stellatus*) and Caspian Sea sprat (*Clupeonella Cultriventris*)) which were in raw form and cooked forms (frying, grilling on charcoal and grilling on the gas oven) was prepared, under the same conditions in the laboratory. These fish samples (in triplicated) were purchased from Mazandaran Province, Iran.

### 2.2 Standards and reagents

For this experiment, biphenyl (using for internal standard), acetonitrile, sodium chloride (NaCl), methanol, potassium hydroxide (KOH), hydrochloric acid, and dichloromethane were bought from Company of Merck (Germany). The Multi Walled Carbon Nanotubes (MWCNTs) as adsorbent was bought from Nanoshel Co. (Panchkula, India), which it diameter was 30–60 nm and it length was 5–30 mm. The MWCNTs-Fe_3_O_4_ was prepared according to the previous study, and the optimal conditions are considered as in the past (Kargarghomsheh, Naghashan, Farhadiyan, Arabameri, Tooryan, & Shariatifar, 2023; Kiani, Ahmadloo, Moazzen, Shariatifar, Shahsavari, Arabameri, et al., 2021; Mehraie, Shariatifar, Arabameri, Moazzen, Mortazavian, Sheikh, et al., 2022; Rezaei, Moazzen, Shariatifar, Khaniki, Dehghani, Arabameri, et al., 2021). The PAH reference standards (order number: CRM47930, QTMPAH-Mix, 2000 Âµg per milliliters) were obtained from Company of Supelco (Bellefonte, PA, USA).

### 2.3 Blank sample preparation

One kilogram of fish was ground twice (minced fish), divided into 100 g pieces and each piece was microwaved for 5 min at a frequency of 2450 MHz with an output power of 900 W. The suitability of this method for blank samples has been confirmed by previous research (Kiani et al., 2021, Mehraie et al., 2022, Rezaei et al., 2021).

### 2.4 Standards preparation

In our study, internal standard, the standard of stock and standard of mixed were prepared according to the prior researches (Kiani et al., 2021, Kiani et al., 2019, Shariatifar et al., 2022) that these standard solutions were kept at 4 °C until experiments.

### 2.5 Samples preparation

The fish meat samples were prepared in four stages including; 1) sample cleanup, 2) adsorption of analytes from food matrices with adsorbent, 3) desorption of analytes from the magnetic adsorbent with dichloromethane solvent, and 4) injecting 1 µ solution (containing analytes) into the GC/MS equipment, by GC syringe) that were described in previous studies (Moazzen et al., 2022, Shariatifar et al., 2021, Shariatifar et al., 2022).

### 2.6 Instrumental analysis

In present study, the GC apparatus (Agilent 6890) was applied that it detector was mass model Agilent 5973 (Palo Alto, CA, United States). Other characteristics and conditions (like carrier gas, temperature of injector and oven, column type, etc.) were according prior researches (Moazzen et al., 2022, Shariatifar et al., 2021, Shariatifar et al., 2022). By the SIM (selective ion monitoring) mode, the PAH compounds were quantified. Based on the Table S1, the experiment qualification was performed by comparing the retention times to reference times of retention under comparable GC–MS conditions using injecting standards of calibration.

### 2.7 Optimization of method

For this purpose, 5 mL of solution of mixed working standard (0.5 μg / mL) was added to the blank sample (500 g). After that, the mixture was homogenized for 30 min and the mixture was stored for 24 h at 4 °C. Finally, this mixture was applied for optimization of technique. Finally, based on the “one factor at a time” test, the method was optimized (Kiani et al., 2019, Shariatifar et al., 2021, Shariatifar et al., 2022).

### 2.8 Validation of the analytical method

Table 2S presents, the linear range, coefficient of determination, LODs, LOQs and recovery percent varied from 0.050 to 20.000 μg/kg, 0.987 to 0.996, 0.1–0.63 μg/kg, 0.3–1.89 μg/kg and 93.7 to 102.6% respectively. An evaluation of the accuracy of method was conducted based on the interday precision via analysis samples of QC (quality control) that were evaluated multiple times over 3 days (consecutive), with 4 levels. The precision percent of all PAHs was<8.8 % for values of inter-day and the percent of reliability was assessed varied from 4.8 to 10.9%.

### 2.9 Characterization of human health risk

#### 2.9.1 EDI (Estimated daily Intake) and non-carcinogenic risk evaluation

The ILCR is an approach to evaluate the risk compounds, which exhibited carcinogenic effects. The ingestion of daily and ILCR value of PAH compounds via the fish ingestion were assessed by 1 and 2 equations according to the previously introduced techniques (Shariatifar, Moazzen, Arabameri, Moazzen, Khaniki, & Sadighara, 2021):

(1) $$\mathrm{BEC}=\sum_{i=1}^{n}C_{i}\times TEF$$

(2) $$EDI\;=\;\frac{C\;\times \;IR\;i\times \;EDi\;\times \;EFi}{BW\;\times \;AT}$$

The illustration of the above parameters are presented in Table 1. The concentration of PAH analytes was converted to benzo (a) pyrene equivalent concentration (BEC; μg/kg) by TEFs (toxicity equivalency factors) (Table s3.) The incremental lifetime cancer risk associated with PAHs (BaP) from group 2A, a human carcinogen (USEPA. Risk assessment guidance for Superfund: volume III part A & risk assessment. Washington), through fish is methodology to calculate the risk and were measured by using Equation 3,4 and 5:

**Table 1:** Variables employed in the current research for estimation of human health exposure in fish.

exposure parameters		unit	Reference
IR	Average daily foods intake (means based on manufactory order)	Kg/day	(Kalantari, 2015)
C	PAHs concentrations	μg/Kg	–
SF	carcinogenic slope factor of oral intake(7.3 per mg/kg/d)	(mg (kg d^–1^))^−1^	(USEPA. Risk assessment guidance for Superfund: volume III part A & risk assessment. Washington)
EFi	exposure frequency	days per year	(Karimi, Shariatifar, Rezaei, Alikord, & Arabameri, 2021)
EDI	estimated daily intake	mg/kg	
AT	average time	days	(Eghbaljoo-Gharehgheshlaghi, Shariatifar, Arab, Alizadeh-Sani, sani, Asdagh, et al., 2020)
ED	exposure duration	days	(Roudbari, Nazari, Shariatifar, Moazzen, Abdolshahi, Mirzamohammadi, et al., 2021)
BW	body weight (70)	kg	(Shariatifar, Seilani, Jannat, Nazmara, & Arabameri, 2020)
BEC	BaP equivalents concentrations by toxicity equivalency factors (TEFs).	---	(EPA, 2011)

**Table 2:** PAHs concentration in fish samples (µg/kg).

	Nap	Acy	Ace	Fle	Ph	An	Fla	Py	BaA	Chr	BbF	BkF	BaP	DahA	BgP	IcdP	Total PAHs	PAH 4
Mean	5.85	0.34	0.70	1.49	0.34	0.25	1.51	2.08	1.31	1.14	1.31	0.80	1.08	1.34	0.32	0.45	20.31	4.85
Median	5.80	0.31	0.72	1.44	0.31	0.19	1.35	2.00	1.22	1.06	1.22	0.84	1.00	1.27	0.25	0.32	19.42	4.58
Min	3.06	0.13	0.16	0.73	0.14	0.08	0.92	1.43	0.76	0.68	0.77	0.24	0.61	0.67	0.25	0.32	11.28	2.88
Max	9.60	0.78	1.43	2.28	0.86	0.67	2.57	3.10	2.08	1.79	2.05	1.33	1.89	2.12	0.64	0.77	33.90	7.81
SD	2.11	0.22	0.38	0.46	0.22	0.17	0.45	0.46	0.37	0.32	0.39	0.29	0.36	0.43	0.14	0.19	6.60	1.40
*p*-value	0.01	0.01	0.00	0.00	0.14	0.02	0.01	0.03	0.02	0.01	0.02	0.00	0.03	0.01	0.38	0.55	0.01	0.01

ILCR = EDI × SF (3)

(4) $$\mathrm{ILCR}=BEC\times EF\times E_{D}\times SF/BW\;\times AT$$

(5) $$\mathrm{ILCR}=\;\frac{BEC\times EF\times ED\times SF}{BW\;\times \;AT}$$

The illustration of parameters are presented in Table 1.

### 2.10 Statistical analyses

The measurement of all data was analyzed by the SPSS statistical packages (version 22). The results were indicated that in three repetitions and were indicated as mean ± standard deviation (SD). Owing to the data non-normality, the test of Mann-Whitney was employed to evaluate the statistical significance of samples. In this study, at *p* < 0.05, the level of significance was assessed.

## 3 Results and discussion

### 3.1 Validation of the analytical method

By comparing the results of the present method (MSPE/GC–MS) with other studies, it can be pointed out that the present method is acceptable (M. M. Hasan, Kubra, Hasan, Awual, Salman, Sheikh, et al., 2023; M. N. Hasan et al., 2023, Khalili et al., 2023, Magalhães et al., 2022, Salman et al., 2023, Salman et al., 2021, Salman et al., 2023, Shariatifar et al., 2022).

### 3.2 Level of PAH compounds in all fish samples

Table 2, shows the maximum (max), median, minimum (min), mean, and standard deviation (SD) of PAH analytes in fish samples. The results indicated that in samples the mean of entire PAHs was 20.31 ± 6.60 µg/kg. The meean of PAH4 in all fish samples was 4.58 ± 1.40 µg/kg that was lower than the EU level of standard for PAH4 in fish (12 µg/kg). The mean of BaP compound in fish samples was 1.08 ± 0.36 µg/kg and varied from 0.61 to 1.89 µg/kg that was lower than the stated level of standard for BaP in fish (2 µg/kg). Nap had the highest concentration between PAHs analytes (5.85 µg/kg) and An had minimum concentration between PAHs analytes (0.25 µg/kg) in all fish samples.

Comparison of the outcomes of the current research with other researches:

Nwaichi et al. assessed PAH compounds in 3 commonly consumed fish and seafood, which reported the average levels of PAH analytes in the mentioned samples ranged from nd to 171.000 ± 0.430 μg/kg wet weight in *Periophthalmus koeleuteri*, nd to 87.400 ± 0.030 μg/kg wet weight in *Crassostrea virginica* and nd to 22.400 ± 0.050 μg/kg wet weight in *Littorina littorea*, which were higher than findings of this research (Nwaichi & Ntorgbo, 2016). Ferrante et al. evaluated 16 PAHs in 3 fish and seafood (*Donax trunculus*, *Solea solea* and *Sardina pilchardus*) from Italy and stated that the mean of 16 PAHs, PAH4 and BaP were ranged from 27.52 to 247.4, 2.558 to 4.620 and 0.296 to 22.59 μg/kg wet weight that mean of 16 PAHs was higher than findings of this study (Ferrante, et al., 2018). Diop et al. in France assessed 4 PAHs in fish and seafood (that including *Penaeus kerathurus*, *Perna perna*, *Solea senegalensis*, *Mugil cephalus*, tilapia (*Sarotherodon melanotheron*), sardinella (*Sardinella aurita*)) and reported that the mean of PAH4 ranged from 1 to 19 µg/kg wet weight, which was lower than our findings in some samples (Diop, Net, Howsam, Lencel, Watier, Grard, et al., 2017). In Spain, Rodríguez-Hernández et al. analyzed the 16 PAHs in fish and seafood including; 1) wild-caught whitefish: parrot fish (*Sparisoma cretense*), megrim (*Stephanoiepis hispidus*), hake (*Merluccius merluccius*), wreckfish (*Polyprion americanus*), seabass (*Dicentrarchus labrax*), sole (*Solea vulgaris*), and toothed sparus (*Dentex dentex*); 2) aquaculture whitefish: gilt head fish (*Sparus aurata*), seabass (*Dicentrarchus labrax*), iridiscent shark (*Pangasius hypophthalmus*), and sole (*Solea vulgaris*); 3) aquaculture bluefish were: trout (*Salmo trutta*) and salmon (*Salmo salar*); 4) wild-caught bluefish were: sardine (*Sardina pilchardus*), salmon (*Salmo salar*), and tuna (*Thunnus thynnus*); 5) different species of seafood, from aquaculture: mussel (*Mytilus galloprovincialis*) and crayfish (*Penaeus* spp.); 6) from wild origin: mussel (*Mytilus galloprovincialis*), crayfish (*Penaeus* spp.), and shrimp (*Parapenaeus* spp.)) and reported that the mean of 16 PAHs and BaP were 0.49–4.32 and n.d. µg/kg wet weight, which were lower than this study results (Rodríguez-Hernández, Camacho, Henríquez-Hernández, Boada, Valerón, Zaccaroni, et al., 2017). Moon et al. (2010) measured the16 PAHs in 26 types of seafood in Korean diets including; 1) eighteen species of fish: rockfish, hairtail, anchovy, Alaska pollack, eel, herring, yellow croaker, cod, mackerel, tuna, ray, Spanish mackerel, angler fish, olive flounder, saury, catfish, flounder, and filefish; 2) four species of bivalve (ark shell, clam, mussel, and oyster); 3) two species of crustacean (shrimp and crab); 4) two species of cephalopod (octopus and squid) and stated the average of sixteen PAH compounds was 30.9 ± 9.76–133 ± 106 µg/kg dry weight, which was higher than our results (Moon, Kim, Choi, & Choi, 2010). In 2022, Ju et al. (in Taiwan) analyzed the 16 PAHs in shellfish (*Corbicula fluminea Formosa* and *Meretrix lusoria*) and aquaculture farmed fish (*Mugil cephalus* and *Oreochromis mossambicus*), and reported that the minimum and maximum levels of total PAHs (among all species) were assessed in. M. *lusoria* (20.0 ± 5.8. µg/kg wet weight) and C. *fluminea Formosa* (43.0 ± 11.3 µg/kg wet weight), respectively, which compared to the results of this research, two cases were more and one case was less (Ju, Chen, Wang, Chen, & Dong, 2022). In 2019, Habibullah-Al-Mamun et al. assessed sixteen PAHs in two species of shellfish and five species of finfish (that the species of shellfish included shrimp crab (*Scylla serrata*) and (*Penaeus indicus*), and the species of finfish included Loitta (*Harpadon nehereus*), Ilish (*Tenualosa ilisha*), Sole (*Cynoglossus lingua*), Poa (*Otolithoides pama*,), and Rupchanda (*Pampus argentius*)) from Bangladesh and reported that the total levels of PAH compounds in the mentioned samples was 117.9–4216.8 µg/kg wet weight in summer and 184.5–2806.6 µg/kg wet weight, in winter, which were higher than our results (Habibullah-Al-Mamun, Ahmed, Islam, Tokumura, & Masunaga, 2019). In Korea, Lee et al. (2015) measured the 8 PAHs from frequently consumed seafood and stated that the mean concentration of BaP was 0.34 µg/kg (which was lower than the result of present study) and the mean total of PAHs concentration was 1.06 µg/kg wet weight (Lee, Lee, & Shin, 2015). In 2021, Udofia analyzed the PAHs levels in 3 seafood species (economically important), including; periwinkles (*Tympanotonus fuscatus*), prawns (*Macrobrachium macrobrachium*), and catfish (Chrysichthys nigrodigitatus) from Nigeria and stated that the mean of total PAH compounds ranged from 4500 to 6360 µg/kg wet weight, in catfish, 4610 to 7750 µg/kg wet weight, in prawns, and 4910 to 6140 µg/kg wet weight, in periwinkles, which were higher than our results (Udofia, Ameh, Miller, & Ekpenyong, 2021). Qian et al. (2022) measured PAHs in a diversity of coastal marine seafood from China and reported that the ƩPAHs ranged from 320 − 2500 µg/kg dry weight, which was higher than our results (Qian, et al., 2022). In Brazil, Magalhães et al. (2022) evaluated PAHs in fishery resources and reported that the total PAHs in the edible tissues of species of shellfish and 34 spices of finfish ranged from 8.71 to 418 µg/kg wet weight, which was lower than our results in some cases (Magalhães, Carreira, Rosa Filho, Rocha, Santana, & Yogui, 2022). In 2013, Drabova et al. assessed PAHs in seafood and canned fish products in oil including; 1) smoked seafood in oil (mussel (*Mytilus edulis*), squid (*Lolig*o species) and octopus (*Octopoda* species)); 2) smoked spices of fish (sardine in oil (*Sardina pilchardus*), smoked herring in oil (*Clupea harengus*), mackerel in oil (*Scomber scombrus*), Baltic sardine in oil (*Sprattus sprattus*) smoked sprats in oil (*Sprattus sprattus*), and smoked mackerel in oil (*Scomber scombrus*)) and stated that the mean of 16 PAHs, PAH4 and BaP were ranged from 1.41 to 116, 0.12 to 54.7 and 0.12 to 9.13 µg/kg, respectively, which in some samples were higher and in others lower than the results of this research (Drabova, Pulkrabova, Kalachova, Tomaniova, Kocourek, & Hajslova, 2013). In Iran, Jafarabadi et al. (2020) measured PAHs in three species of fish (including S. *guttatus*, L. *microdon* and L. *argentimaculatus*) and reported that the concentrations of ∑PAHs were 1390 and 1004 µg/kg freeze-dried weight for muscle and liver, respectively, which was higher than our results (Jafarabadi, Mashjoor, Bakhtiari, & Jadot, 2020).

The amount of fish PAHs contamination can depend on many factors such as the amount of environmental pollution (water, air and soil), the life span of the fish, the weight of the fish, the type of fish (farmed or wild), the type of food consumed by the fish, fish preparation methods (raw or processed), as well as contamination during fishing (surface pollution, etc.). It can also be mentioned that different cooking methods can affect the amount of pH depending on time, temperature and cooking conditions. Even sometimes additives added to fish such as spices and other compounds can increase the level of contamination (Drabova et al., 2013, Habibullah-Al-Mamun et al., 2019, Magalhães et al., 2022, Moon et al., 2010, Nwaichi and Ntorgbo, 2016).

### 3.3 The impact of the different types of fish on the amount of PAHs contamination

Table 3, shows the median, min, mean, max, and ± SD of PAH analytes in different types of fish samples (carp, Caspian Kutum, Caspian Sea sprat, starry sturgeon and trout). The results presented Starry Sturgeon had mean highest of total PAH compounds (13.24 ± 1.84 µg/kg) and Caspian Sea sprat had mean lowest of total PAH compounds (1.24 ± 0.8 µg/kg), which the total rank of ƩPAH analytes in the 5 groups of fish with different types was starry sturgeon > carp > Caspian Kutum > trout > Caspian Sea sprat, which can probably be associated to the age of different fishes, weight and size of different fishes (that is, the higher the age and weight of the fish, the higher the level of contamination and vice versa). The mean lowest of BaP compound was in Caspian Sea sprat fish (0.78 ± 0.09 µg/kg) and highest average of it was in Starry Sturgeon fish (1.58 ± 0.39 µg/kg), which were lower than the EU level of standard. Furthermore, the mean level of PAH4 was highest and lowest in Caspian Sea sprat fish (3.44 ± 0.55 µg/kg) and Starry Sturgeon fish (6.70 ± 1.08 µg/kg), respectively, which were lower than the stated level of standard.

**Table 3:** The impact of the different types of fish on the amount of PAHs contamination (µg/kg).

		Nap	Acy	Ace	Fle	Ph	An	Fla	Py	BaA	Chr	BbF	BkF	BaP	DahA	BgP	IcdP	Total PAHs	PAH 4
Carp	Mean	7.29	0.53	0.99	1.79	0.38	0.31	1.73	2.31	1.41	1.26	1.45	0.94	1.19	1.64	0.43	0.53	24.15	5.31
	Median	7.22	0.58	0.95	1.80	0.35	0.29	1.69	2.33	1.30	1.23	1.31	0.93	1.15	1.69	0.43	0.51	23.57	4.95
	Min	5.69	0.30	0.80	1.56	0.14	0.19	1.35	1.93	1.19	0.97	1.20	0.80	0.97	1.17	0.25	0.32	19.19	4.39
	Max	9.02	0.65	1.25	2.00	0.70	0.48	2.18	2.66	1.85	1.60	2.00	1.11	1.50	1.99	0.60	0.77	30.29	6.95
	SD	1.37	0.16	0.19	0.18	0.23	0.12	0.34	0.37	0.30	0.26	0.37	0.13	0.22	0.35	0.20	0.24	4.59	1.13
Caspian Kutum	Mean	6.13	0.30	0.80	1.47	0.36	0.22	1.50	1.88	1.34	1.19	1.38	0.88	1.01	1.34	0.34	0.56	20.69	4.92
	Median	5.97	0.31	0.76	1.48	0.31	0.19	1.34	1.74	1.35	1.20	1.24	0.88	0.97	1.29	0.25	0.67	19.65	4.76
	Min	4.20	0.13	0.65	1.25	0.14	0.08	1.29	1.65	1.05	0.95	1.11	0.72	0.85	1.00	0.25	0.32	15.94	3.96
	Max	8.23	0.45	1.00	1.68	0.64	0.40	1.88	2.24	1.63	1.42	1.79	1.03	1.20	1.72	0.51	0.68	26.49	6.04
	SD	2.02	0.16	0.18	0.22	0.25	0.16	0.33	0.32	0.29	0.24	0.36	0.16	0.18	0.36	0.15	0.21	5.35	1.05
Caspian Sea Sprat	Mean	3.48	0.17	0.16	0.89	0.19	0.13	1.09	1.77	0.92	0.81	0.94	0.43	0.78	0.94	0.25	0.32	13.24	3.44
	Median	3.38	0.13	0.16	0.90	0.14	0.13	1.06	1.77	0.86	0.79	0.94	0.49	0.78	0.95	0.25	0.32	13.01	3.36
	Min	3.06	0.13	0.16	0.73	0.14	0.08	0.92	1.52	0.76	0.68	0.77	0.24	0.67	0.86	0.25	0.32	11.28	2.88
	Max	4.11	0.28	0.16	1.03	0.32	0.18	1.34	2.00	1.19	0.98	1.11	0.52	0.89	1.00	0.25	0.32	15.67	4.17
	SD	0.45	0.08	0.00	0.12	0.09	0.06	0.18	0.23	0.19	0.13	0.15	0.13	0.09	0.06	0.00	0.00	1.84	0.55
Starry Sturgeon	Mean	8.24	0.60	1.12	2.11	0.56	0.48	2.05	2.65	1.80	1.56	1.77	1.17	1.58	1.81	0.35	0.50	28.34	6.70
	Median	8.25	0.63	1.04	2.10	0.53	0.46	2.07	2.64	1.76	1.60	1.83	1.20	1.70	1.87	0.25	0.49	28.35	6.87
	Min	6.87	0.34	0.98	1.98	0.30	0.35	1.51	2.23	1.60	1.24	1.37	0.95	1.03	1.38	0.25	0.32	22.75	5.24
	Max	9.60	0.78	1.43	2.28	0.86	0.67	2.57	3.10	2.08	1.79	2.05	1.33	1.89	2.12	0.64	0.71	33.90	7.81
	SD	1.14	0.19	0.21	0.12	0.25	0.14	0.45	0.38	0.23	0.23	0.29	0.16	0.39	0.32	0.20	0.22	4.64	1.08
Trout	Mean	4.36	0.18	0.48	1.19	0.22	0.15	1.23	1.75	1.15	0.95	1.11	0.62	0.95	1.02	0.25	0.42	16.02	4.16
	Median	3.89	0.13	0.43	1.22	0.14	0.12	1.20	1.74	1.17	0.98	1.11	0.55	0.98	1.01	0.25	0.32	15.17	4.19
	Min	3.75	0.13	0.40	1.00	0.14	0.08	1.06	1.43	0.86	0.77	0.85	0.49	0.76	0.67	0.25	0.32	13.05	3.34
	Max	5.90	0.31	0.67	1.34	0.47	0.28	1.47	2.10	1.41	1.08	1.36	0.88	1.10	1.38	0.25	0.72	20.71	4.94
	SD	1.03	0.09	0.13	0.14	0.17	0.10	0.17	0.35	0.23	0.13	0.21	0.18	0.17	0.30	0.00	0.20	3.28	0.67
*p*-value		0.01	0.01	0.00	0.00	0.14	0.02	0.01	0.03	0.02	0.01	0.02	0.00	0.03	0.01	0.38	0.55	0.01	0.01

### 3.4 The impact of different methods of cooking on the concentration of PAHs contamination in fish samples

Table 4, is shown the impact of different methods of cooking on the PAH concentrations in fish samples. The present results showed that the raw fish samples had the lowest amount of PAHs and different cooking methods increased the amount of these compounds in the fish samples. Also, the findings stated that any cooking method can increase the amount of these compounds according to the temperature, time and conditions of cooking. The mean highest of entire PAHs and PAH4 level were seen in grilled fish on charcoal (25.41 ± 7.31 and 5.98 ± 1.47 µg/kg, respectively) and mean lowest of them were seen in raw fish samples (16.44 ± 4.63 and 3.96 ± 0.92 µg/kg, respectively). Furthermore, the minimum and maximum mean of BaP identified in grilled fish on gas oven and grilled fish on charcoal, respectively. The total rank of PAH analytes in the four groups of fish with different cooking methods was grilled fish on charcoal > fried fish > grilled fish on gas oven > raw fish. Our results show that processing increases the contamination rate compared to raw fish, in the meantime, the fish cooked on coal and fried had higher pollution, which can probably be due to the increase of pollution transferred from coal and oil to the fish (Gorji et al., 2016, Shariatifar et al., 2021, Shariatifar et al., 2022).

**Table 4:** The impact of different methods of cooking on the PAH concentrations in fish samples (µg/kg).

		Nap	Acy	Ace	Fle	Ph	An	Fla	Py	BaA	Chr	BbF	BkF	BaP	DahA	BgP	IcdP	Total PAHs	PAH 4
Raw fish	Mean	4.71	0.21	0.60	1.30	0.21	0.16	1.30	1.77	1.09	0.92	1.07	0.64	0.88	1.02	0.25	0.32	16.44	3.96
	Median	4.20	0.13	0.65	1.25	0.14	0.08	1.34	1.74	1.05	0.95	1.11	0.72	0.86	1.00	0.25	0.32	15.94	3.96
	Min	3.06	0.13	0.16	0.73	0.14	0.08	0.92	1.43	0.76	0.68	0.77	0.24	0.67	0.67	0.25	0.32	11.28	2.88
	Max	6.87	0.34	0.98	1.98	0.31	0.40	1.65	2.23	1.60	1.24	1.37	0.95	1.03	1.38	0.25	0.32	22.75	5.24
	SD	1.54	0.11	0.33	0.49	0.09	0.14	0.30	0.32	0.33	0.22	0.26	0.28	0.14	0.27	0.00	0.00	4.63	0.92
Grilled fish on charcoal	Mean	7.37	0.49	0.90	1.67	0.60	0.40	1.89	2.41	1.63	1.37	1.66	0.97	1.31	1.64	0.45	0.64	25.41	5.98
	Median	8.23	0.45	1.00	1.68	0.64	0.40	1.88	2.24	1.63	1.42	1.79	1.03	1.20	1.72	0.51	0.71	26.49	6.04
	Min	4.11	0.28	0.16	1.03	0.32	0.18	1.34	2.00	1.19	0.98	1.11	0.52	0.89	1.00	0.25	0.32	15.67	4.17
	Max	9.60	0.78	1.43	2.28	0.86	0.67	2.57	3.10	2.08	1.79	2.05	1.33	1.89	2.12	0.64	0.77	33.90	7.81
	SD	2.30	0.22	0.51	0.50	0.21	0.19	0.51	0.45	0.35	0.34	0.41	0.30	0.39	0.46	0.19	0.18	7.31	1.47
Grilled fish on gas oven	Mean	5.46	0.33	0.63	1.48	0.26	0.25	1.40	2.03	1.20	1.12	1.23	0.78	0.98	1.34	0.25	0.39	19.12	4.53
	Median	5.15	0.13	0.61	1.39	0.29	0.18	1.20	2.00	1.13	1.03	1.09	0.80	0.76	1.25	0.25	0.32	17.13	3.98
	Min	3.31	0.13	0.16	0.87	0.14	0.08	1.00	1.62	0.83	0.76	0.87	0.45	0.61	0.98	0.25	0.32	12.62	3.22
	Max	7.95	0.70	1.00	2.10	0.41	0.51	1.93	2.47	1.62	1.61	1.86	1.18	1.59	1.76	0.25	0.70	27.26	6.68
	SD	1.99	0.28	0.37	0.49	0.12	0.17	0.40	0.30	0.30	0.31	0.39	0.30	0.40	0.33	0.00	0.17	6.02	1.38
Fried fish	Mean	5.86	0.35	0.67	1.50	0.29	0.19	1.44	2.10	1.31	1.17	1.27	0.82	1.16	1.37	0.32	0.46	20.28	4.91
	Median	5.97	0.31	0.76	1.48	0.14	0.19	1.29	1.92	1.24	1.20	1.20	0.88	1.10	1.29	0.25	0.32	19.65	4.76
	Min	3.45	0.13	0.16	0.92	0.14	0.08	1.11	1.47	0.88	0.82	1.00	0.52	0.80	0.90	0.25	0.32	13.41	3.50
	Max	8.55	0.60	1.07	2.09	0.65	0.35	2.20	2.80	1.89	1.58	1.79	1.22	1.80	1.98	0.60	0.68	29.45	7.06
	SD	2.20	0.23	0.37	0.46	0.23	0.12	0.44	0.60	0.37	0.29	0.30	0.28	0.38	0.49	0.16	0.20	6.54	1.31
*p*-value		0.22	0.24	0.56	0.68	0.03	0.18	0.24	0.14	0.09	0.15	0.16	0.33	0.16	0.17	0.07	0.04	0.21	0.16

### 3.5 Probabilistic assessment of health risk

Exposure to low concentrations of PAHs can lead to health problems for consumers. In adults who ingest fish, the rank order of PAHs based on their EDI was Py > Ph > Nap > IcdP > Fle > Fla > DahA > Chr > BkF > BgP > BbF > BaP > BaA > An > Acy > Ace. According to the World Health Organization (WHO), daily exposure to dietary BaP above 10 ng/kg bw day^−^ is hazardous to human health. However, the values of EDI for fish samples in this study were below 1, indicating that the levels of PAHs in the fish samples were not dangerous to the public's health. (Table 5). These results are consistent with a previous report by Tien, Bogdanović et al. (2019) who estimated the dietary exposure of PAH compounds in fish and meat in Croatia (Bogdanović, Pleadin, Petričević, Listeš, Sokolić, Marković, et al., 2019).

**Table 5:** The EDI in the adults and children due to fish content of PAH ingestion.

EDI	adults				children			
Percentiles	5%	50%	75%	95%	5%	50%	75%	95%
Nap	3.75E-4	5.49E-4	6.51E-4	8.25E-4	1.73E-3	2.61E-3	3.09E-3	3.87E-3
Acy	2.03E-5	2.96E-5	3.49E-5	4.39E-5	9.11E-5	1.36E-4	1.62E-4	2.08E-4
Ace	4.58E-5	6.80E-5	8.01E-5	1.02E-4	2.16E-4	3.17E-4	3.73E-4	4.76E-4
Fle	9.21E-5	1.37E-4	1.61E-4	2.05E-4	4.30E-4	6.35E-4	7.48E-4	9.51E-4
Ph	1.97E-5	2.94E-5	3.44E-5	4.46E-5	9.19E-5	1.35E-4	1.64E-4	2.04E-4
An	1.23E-5	1.80E-5	2.13E-5	2.73E-5	5.48E-5	8.37E-5	9.92E-5	1.27E-4
Fla	8.57E-5	1.29E-4	1.52E-4	1.90E-4	3.96E-4	6.00E-4	7.11E-4	9.14E-4
Py	1.31E-4	1.91E-4	2.22E-4	2.77E-4	5.91E-4	8.93E-4	1.05E-3	1.37E-3
BaA	7.55E-5	1.15E-4	1.36E-4	1.79E-4	3.69E-4	5.41E-4	6.26E-4	8.00E-4
Chr	6.72E-5	1.03E-4	1.21E-4	1.53E-4	3.21E-4	4.69E-4	5.54E-4	7.11E-4
BbF	7.67E-5	1.15E-4	1.37E-4	1.75E-4	3.62E-4	5.49E-4	6.38E-4	8.07E-4
BkF	5.48E-5	8.14E-5	9.44E-5	1.21E-4	2.49E-4	3.73E-4	4.41E-4	5.57E-4
BaP	6.37E-5	9.44E-5	1.10E-4	1.39E-4	3.02E-4	4.47E-4	5.17E-4	6.68E-4
DahA	8.08E-5	1.22E-4	1.44E-4	1.85E-4	3.79E-4	5.71E-4	6.76E-4	8.36E-4
BgP	1.59E-5	2.37E-5	2.81E-5	3.65E-5	7.29E-5	1.10E-4	1.30E-4	1.65E-4
IcdP	2.03E-5	3.03E-5	3.54E-5	4.48E-5	9.20E-5	1.41E-4	1.64E-4	2.13E-4

**Fig. 1:**
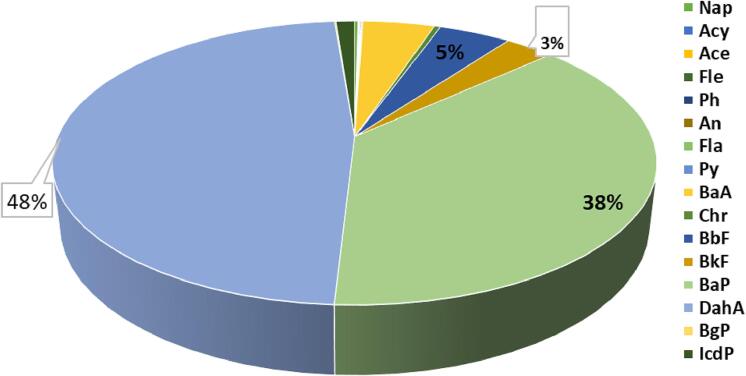
Value of BEC and contribution owing to PAHs content in fish.

The equivalent concentration of BaP for all PAH analytes was 2.65 μg/kg. Fig. 1 shows that the DahA and BaP are the two main types of PAHs in the total BEC (μg/kg), while the the other PAHs had a portion of less than fourteen percent.

The Monte Carlo simulations can be used in risk assessment efforts to enable safety professionals to select better decisions, provide probabilistic estimates for placing risks into the matrix or plot based on the probability and intensity of a potential event, recognize hazard-producing process stages driving undesirable variation, predict food quality in later phases of the process, and product changes, etc. The best examples of this research are studies about baby food (Moazzen, Shariatifar, Arabameri, Hosseini, & Ahmadloo, 2022), edible mushrooms (Shariatifar, Moazzen, Arabameri, Moazzen, Khaniki, & Sadighara, 2021), yogurt and butter (Kiani, et al., 2021), tea and coffee samples (Roudbari, et al., 2021), non-dioxin like-polychlorinated biphenyls (NDL-PCBs) in samples of butter (Yaminifar, Aeenehvand, Ghelichkhani, Ahmadloo, Arabameri, Moazzen, et al., 2021), milk and milk powder (Shariatifar, Dadgar, Fakhri, Shahsavari, Moazzen, Ahmadloo, et al., 2020), acrylamide of nuggets (Seilani, Shariatifar, Nazmara, Khaniki, Sadighara, & Arabameri, 2021), pesticide of pistachio (Arabameri, Mohammadi Moghadam, Monjazeb Marvdashti, Mehdinia, Abdolshahi, & Dezianian, 2020),cereal products (Khalili, Shariatifar, Dehghani, Yaghmaeian, Nodehi, Yaseri, et al., 2022), heavy metals in fruit juices (Karami, Shariatifar, Khaniki, Nazmara, Arabameri, & Alimohammadi, 2021), sulfur dioxide of raisins (Saghafi, Shariatifar, Alizadeh Sani, Dogaheh, Khaniki, & Arabameri, 2021). In this study, the ILCR for the adults and children was computed based on Monte Carlo simulation; BaPeq was used as an indicator in the ILCR models. The distribution function and alternative marginal were adjusted by fitting the Monte Carlo parameters. The simulation was run individually for 10,000 iterations to provide stability in the outcome.

**Fig. 2:**
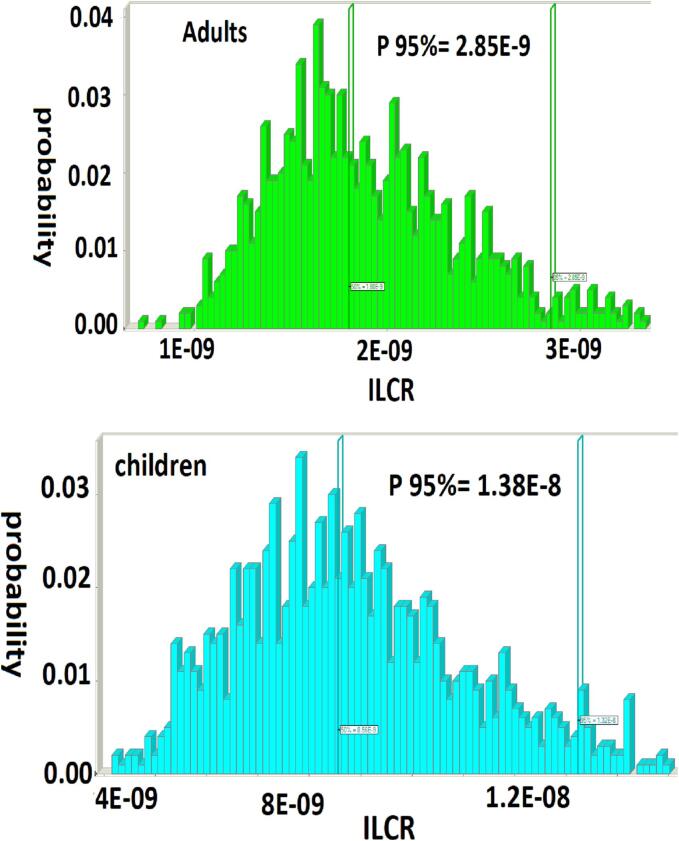
The ILCR in the adults and children due to fish content of PAH ingestion.

In terms of carcinogenic risk, as stated from investigations, the ILCR indexes (percentile 95%) for children and adults due to ingestion of fish were equal to 1.32E-8 and 2.85E-9, respectively (Fig. 2). Therefore, consumers in Iran are not at serious health risk (ILCR < 1 × 10^−4^). Similar findings were reported by Yoon et al. (2007) in Korea, where the ILCR value of PAH compounds via 27 different food types was between 2.03 × 10^−6^ to 2.35 × 10^−5^ (Yoon, Park, Lee, Yang, & Lee, 2007). These results suggest that the estimated levels of PAHs in fish samples in this study are within safe limits for consumption and do not pose a significant health risk to consumers.

## 4 Conclusion

For the first time in Iran and other countries, this study evaluated samples of highly consumed fish (cooked with different methods) in terms of their PAH pollutant content and the associated carcinogenic risk. An MSPE-GC/MS method was developed for the extraction of 16 PAH analytes, which resulted in a clean extraction and recovery of PAH compounds of more than 93.7%. The levels of BaP and PAH4 in the samples were found to be lower than the EU standard level. The total rank of ƩPAH analytes in the 5 groups of fish with different types was found to be starry sturgeon > carp > Caspian Kutum > trout > Caspian Sea sprat. Additionally, the total rank of ƩPAH analytes in the four groups of fish with different cooking methods was grilled fish on charcoal > fried fish > grilled fish on gas oven > raw fish. In terms of carcinogenic, consumers were at an acceptable range (ILCR < 1.00E-6) due to ingestion of fish containing PAHs. The results of the findings of the present study are consistent with other studies, which have shown that the amount of these compounds in different types of fish is different and cooking conditions can also affect the amount of this pollutant in food. Overall, this study suggests that the consumption of fish cooked with various methods does not pose a risk to human health. However, it is recommended to measure PAH compounds in other fish available in Iran as well as in canned fish to ensure the safety of consumers.

## CRediT authorship contribution statement

**Gholamali Sharifiarab:** Writing – original draft, Methodology, Writing – review & editing. **Mohammad Ahmadi:** Conceptualization, Supervision, Methodology, Writing – review & editing, Validation. **Nabi Shariatifar:** Conceptualization, Supervision, Methodology, Writing – review & editing, Validation. **Peiman Ariaii:** Visualization, Investigation, Methodology, Software, Data curation, Validation.

## Declaration of Competing Interest

The authors declare that they have no known competing financial interests or personal relationships that could have appeared to influence the work reported in this paper.

## Data Availability

No data was used for the research described in the article.
