# Supplementary material for: Investigating the effect of type of fish and different cooking methods on the residual amount of polycyclic aromatic hydrocarbons (PAHs) in some Iranian fish: A health risk assessment
Source: Food Chem X. 2023 Jul 8;19:100789. doi: 10.1016/j.fochx.2023.100789 (PMC10534125; doi:10.1016/j.fochx.2023.100789)
Supplement: Supplementary data 1 [file mmc1.docx]

Table S1. GC–MS conditions for the quantification and qualification of PAH analytes

| Ion group | Compounds (PAHs) | Time window  (min.) | Confirmation  ions (m/z) | Quantification  ion (m/z) |
| --- | --- | --- | --- | --- |
| 1 | I.S. (Biphenyl) | 8–13 | 153, 152 | 154 |
| 1 | Acy (Acenaphthylene) | 6–13 | 128, 127 | 128 |
| 1 | Nap (Naphthalene) | 8–13 | 153, 151 | 152 |
| 1 | Ace (Acenaphthene) | 8–13 | 154, 152 | 153 |
| 2 | Fle (Fluorene) | 13–15 | 165, 167 | 166 |
| 3 | Ph (Phenanthrene) | 15–17 | 179, 176 | 178 |
| 3 | An (Anthracene) | 15–17 | 179, 176 | 178 |
| 4 | Py (Pyrene) | 17–20 | 203, 101 | 202 |
| 4 | Fla (Fluoranthene) | 17–20 | 203, 101 | 202 |
| 5 | Chr (Chrysene) | 20–23 | 226, 229 | 228 |
| 5 | BaA (Benzo[a] anthracene) | 20–23 | 226, 229 | 228 |
| 6 | BkF (Benzo[k] fluoranthene) | 23–28 | 253, 126 | 252 |
| 6 | BaP (Benzo[a]pyrene) | 23–28 | 253, 126 | 252 |
| 6 | BbF (Benzo[b] fluoranthene) | 23–28 | 253, 126 | 252 |
| 7 | BgP (Benzo[g,h,i] perylene) | 28–31 | 277, 138 | 276 |
| 7 | DahA (Dibenzo[a,h] anthracene) | 28–31 | 279,139 | 278 |
| 7 | IcdP (Indeno[1,2,3– cd]pyrene) | 28–31 | 277,138 | 276 |

**Table S2. The LOD, LOQ, recoveries, linear range, and coefficient of determination (r2).**

| Target compound | Linear range (µg/kg) | Limit of detection (LOD) (µg/L) | Limit of quantification (LOQ) (µg/L) | Coefficient of determination (r2) | Recoveries (%) |
| --- | --- | --- | --- | --- | --- |
| Nap | 0.050-20 | 0.11 | 0.33 | 0.987 | 100.8 |
| Acy | 0.050-20 | 0.26 | 0.78 | 0.990 | 101.6 |
| Ace | 0.050-20 | 0.31 | 0.93 | 0.992 | 98.9 |
| Fle | 0.050-20 | 0.42 | 1.26 | 0.988 | 94.9 |
| Ph | 0.050-20 | 0.28 | 0.84 | 0.994 | 102.4 |
| An | 0.050-20 | 0.15 | 0.45 | 0.998 | 100.5 |
| Fla | 0.050-20 | 0.30 | 0.90 | 0.990 | 102.6 |
| Py | 0.050-20 | 0.18 | 0.54 | 0.990 | 100.5 |
| BaA | 0.050-20 | 0.21 | 0.63 | 0.989 | 101.4 |
| Chr | 0.050-20 | 0.15 | 0.45 | 0.987 | 102.1 |
| BbF | 0.050-20 | 0.16 | 0.48 | 0.996 | 98.8 |
| BkF | 0.050-20 | 0.11 | 0.33 | 0.988 | 93.7 |
| BaP | 0.050-20 | 0.1 | 0.3 | 0.989 | 100.7 |
| DahA | 0.050-20 | 0.31 | 0.93 | 0.990 | 100.8 |
| BgP | 0.050-20 | 0.5 | 1.50 | 0.994 | 102.0 |
| IcdP | 0.050-20 | 0.63 | 1.89 | 0.991 | 96.4 |

Table S3: PAH analytes and their TEFs

| PAHs | TEF | PAHs | TEF |
| --- | --- | --- | --- |
| Benzo(a)pyrene (BaP) | 1 | Anthracene (A) | 0.01 |
| Dibenz(a,h)anthracene (DahA) | 1 | Naphthalene (NA) | 0.001 |
| Benzo(k)fluoranthene (BkF) | 0.1 | Acenaphthylene (AC) | 0.001 |
| Indeno(l,2,3-cd)pyrene (IcdP) | 0.1 | Acenaphthene (ACE) | 0.001 |
| Benz(a)anthracene (BaA) | 0.1 | Phenanthrene (PHE) | 0.001 |
| Benzo(b)fluoranthene (BbF) | 0.1 | Fluorine (FLO) | 0.001 |
| Chrysene (CHR) | 0.01 | Pyrene (PYR) | 0.001 |
| Benzo(g,h,i)perylene (BghiP) | 0.01 | Fluoranthene (FL) | 0.001 |
